# Expression of Pleiotrophin in the Prostate is Androgen Regulated and it Functions as an Autocrine Regulator of Mesenchyme and Cancer Associated Fibroblasts and as a Paracrine Regulator of Epithelia

**DOI:** 10.1002/pros.21244

**Published:** 2010-09-01

**Authors:** Brigid Orr, Griet Vanpoucke, O Cathal Grace, Lee Smith, Richard A Anderson, Antony CP Riddick, Omar E Franco, Simon W Hayward, Axel A Thomson

**Affiliations:** 1 MRC Human Reproductive Sciences Unit, The Queen's Medical Research InstituteEdinburgh, UK; 2 Centre for Reproductive Biology, Reproductive and Developmental Sciences Section, University of Edinburgh, The Queen's Medical Research InstituteEdinburgh, UK; 3 Urology Department, Western General HospitalEdinburgh, UK; 4 Departments of Urologic Surgery and Cancer Biology, Vanderbilt University Medical CenterNashville, Tennessee

**Keywords:** prostate, prostate development, mesenchyme, stroma, stromal/epithelial interactions, cancer associated fibroblasts, pleiotrophin, androgens

## Abstract

**BACKGROUND:**

Androgens and paracrine signaling from mesenchyme/stroma regulate development and disease of the prostate, and gene profiling studies of inductive prostate mesenchyme have identified candidate molecules such as pleiotrophin (Ptn).

**METHODS:**

Ptn transcripts and protein were localized by in situ and immunohistochemistry and Ptn mRNA was quantitated by Northern blot and qRT-PCR. Ptn function was examined by addition of hPTN protein to rat ventral prostate organ cultures, primary human fetal prostate fibroblasts, prostate cancer associated fibroblasts, and BPH1 epithelia.

**RESULTS:**

During development, *Ptn* transcripts and protein were expressed in ventral mesenchymal pad (VMP) and prostatic mesenchyme. Ptn was localized to mesenchyme surrounding ductal epithelial tips undergoing branching morphogenesis, and was located on the surface of epithelia. hPTN protein stimulated branching morphogenesis and stromal and epithelial proliferation, when added to rat VP cultures, and also stimulated growth of fetal human prostate fibroblasts, prostate cancer associated fibroblasts, and BPH1 epithelia. *PTN* mRNA was enriched in patient-matched normal prostate fibroblasts versus prostate cancer associated fibroblasts. *PTN* also showed male enriched expression in fetal human male urethra versus female, and between wt male and ARKO male mice. Transcripts for *PTN* were upregulated by testosterone in fetal human prostate fibroblasts and organ cultures of female rat VMP. Ptn protein was increased by testosterone in organ cultures of female rat VMP and in rat male urethra compared to female.

**CONCLUSIONS:**

Our data suggest that in the prostate Ptn functions as a regulator of both mesenchymal and epithelial proliferation, and that androgens regulate Ptn levels. *Prostate 71:305–317, 2011*. © 2010 Wiley-Liss, Inc.

## INTRODUCTION

Stromal–epithelial interactions are involved in the regulation of prostate organogenesis and tumorigenesis. During prostate development, androgen action in mesenchyme regulates proliferation and differentiation of the prostatic epithelium, and in turn the epithelium regulates differentiation of the mesenchyme. Importantly, paracrine signaling from the mesenchyme to the epithelium acts to (1) specify prostatic epithelium identity, (2) induce bud formation, (3) elicit prostatic bud growth and ductal branching, (4) promote differentiation of secretory epithelia, and (5) specify the types of secretory proteins that are expressed, reviewed in Ref. 1. This suggests that mesenchyme is the source of potent paracrine factors, yet few studies have endeavored to directly identify them 2,3. The stroma is also involved in neoplastic prostate growth (reviewed in Ref. 4) and cancer associated fibroblasts (CAFs) exhibit a variety of functional differences relative to normal stroma (reviewed in 5). Tumor stroma is no longer able to restrain prostatic epithelial proliferation, but instead CAFs stimulate tumor growth 6,7 and angiogenesis 8.

To determine which molecules and pathways are active within developmental mesenchyme, we performed serial analysis of gene expression (SAGE) gene profiling of inductive mesenchyme of the ventral mesenchymal pad (VMP) 2. This identified mesenchymal expression of pleiotrophin (Ptn) and suggested a localization to the VMP. Fgf10 9, BMP 4 10, Scube1 2, Notch2, Dlk1, 11 and Wnt 5a 3 have previously been shown to be expressed in prostate mesenchyme and most of these have a functional role in prostate organogenesis. Since androgens stimulate prostate organogenesis via the mesenchymal compartment, this has led to the hypothesis that there may be “andromedins” made by the mesenchyme that act upon epithelia and which are produced in response to androgens. However, there is no direct evidence that “andromedins” identified in developing prostate are essential for androgen action, and there is controversy regarding direct or indirect regulation 12,13.

Ptn is a secreted, highly conserved cytokine, and belongs to a novel two member family of heparin binding molecules, sharing structural and functional similarities with midkine 14. Ptn is an 18 kDa protein, also known as heparin affin regulatory peptide (HARP) and heparin-binding growth-associated molecule (HB-GAM). Ptn protein interacts with heparin sulfate proteoglycans (HSPGs) at the extracellular matrix 15. Biological actions of Ptn are reported to be mediated by at least three different receptors; receptor protein tyrosine phosphatase, RPTB/ξ 16, syndecan-3 (*N*-syndecan) 17, and anaplastic lymphoma kinase (ALK) 18. *Ptn* is responsive to regulation by steroid hormones, and *Ptn* transcript levels are upregulated in the presence of dihydrotestosterone or estradiol, in vitro and in vivo, respectively 15,19.

Ptn plays a key role in cellular growth and differentiation and has been implicated as playing an important role during development. *Ptn* expression is present in tissues during late embryogenesis and perinatal growth, and expression usually decreases around the time of birth 20,21. Ptn is most frequently reported in tissues derived from mesoderm and in organs where mesenchymal–epithelial interactions play an important role, such as the salivary gland, lung, pancreas, kidney, and mammary gland 22–24. In branching organs Ptn may also be important for branching morphogensis 25,26. Ptn has not previously been studied in the developing prostate, although it is reported to play a role in prostate cancer 15. *Ptn*-deficient mice have no gross anatomical abnormalities 27 and it has been proposed that *Ptn* and midkine, which are often co-expressed, might compensate for each other 23. Mice lacking both Ptn and midkine show female infertility and reproductive abnormalities though no effects upon male fertility or reproductive anatomy were reported 28.

Ptn has diverse activity in vitro, stimulating the proliferation of a wide range of cells including epithelial, endothelial, and fibroblastic cell lines 29,30, and also stimulates progenitor cells in primary culture to enter lineage-specific differentiation pathways 20,31. Ptn expression is associated with inflammatory diseases and tumor growth progression, and Ptn may recruit stromal tissue and vasculature to tumors 32–35. *Ptn* expression has been observed in prostate, ovarian, testicular, pancreatic and breast cancer, solid gliomas, neuroblastomas, melanomas, and in several malignant cell lines of different origin, reviewed in Ref. 36.

We have examined the expression, distribution and function of Ptn in the developing prostate, as it was previously suggested to show mesenchymal expression 2, and we hypothesized that it may function as a paracrine regulator of prostate development. *Ptn* expression was restricted to VMP and mesenchyme of the VP as well as the smooth muscle of the urethra. Ptn protein was located in the mesenchyme and at the surface of epithelial cells, and displayed a gradient showing the highest levels at the tips of epithelial ducts. Recombinant hPTN increased proliferation and branching morphogenesis in cultures of VP organs grown in vitro, and stimulated the growth of fibroblasts derived from developing prostate and prostate cancer and prostate epithelial cells. Expression of *Ptn* mRNA and protein was increased by androgens in developmental mesenchyme, and showed sexually dimorphic expression.

## MATERIALS AND METHODS

### Isolation and Culture of Primary Human Cells

Human fetal prostate tissue was obtained following medical termination of pregnancy. Consent was obtained in accordance with UK guidelines and the study was approved by the Lothian Research Ethics Committee. The bladder and urethra were excised, and the prostate microdissected prior to either RNA isolation or primary culture of embryonic prostate fibroblasts (hEPFs). Human adult prostate tissue was obtained from patients undergoing surgery at the Western General Hospital, Edinburgh. Primary CAFs were derived from tissue obtained from patients at the time of transurethral resection of the prostate (TURP). Patient consent was obtained prior to surgery and the study was approved by ethical review (MREC 02/5/63). To culture primary stromal cells from embryonic or cancer samples, tissue specimens were cut into small pieces (1 mm × 1 mm × 1 mm), and plated on a flask coated with fetal calf serum (FCS). Primary cells were grown in DMEM supplemented with 10% FCS and 1× penicillin/streptomycin and CAFs were tested for tumorigenic activity in vivo 7.

### Tissue Isolation From Rodents

Prostate and VMP tissues were microdissected from the urogenital tract of Wistar rats and ARKO mice. The day of copulatory plug observation was taken as e0.5, and day of birth was designated P0. Animal procedures were conducted in accordance with UK law regulated by the Home Office. The P0 female bladder and urethra were removed and dissected to isolate the urethra adjacent to the bladder termed the VSU, which comprised the *V*MP, *s*mooth muscle, and *u*rethral epithelia. Additionally, P0 VMP and ventral prostate (VP, from males) were microdissected.

ARKO mice were produced by mating female mice heterozygous for the X-linked hypoxanthine phosphoribosyltransferase-Cre transgene with male ARflox mice 37. This resulted in females carrying one deleted allele and one wild-type (WT) allele of the X-linked AR gene, which were subsequently mated to produce ARKO males and control littermates. ARKO males were identified by examination upon dissection and lacked prostate and Wolffian derived organs.

### RNA Extraction and Quantitative RT-PCR

Total RNA was extracted using the RNeasy™ Mini kit (Qiagen, Crawley, UK) and RNA quality was measured on the Agilent 2100 Bioanlayser (Agilent Biotechnologies, Santa Clara, CA). Quantitative PCR's were performed on ABI 7500 machine, using the PowerSybr PCR mastermix (ABI, Warrington, UK). Transcript abundances were normalized to *tbp*/*TBP* expression. The PCR primer sequences are provided below:

**Table:** 

Gene	Forward	Reverse
Rn *Ptn* NM_017066.2	ACTGGAAGAAGCAGTTTGGACC	GAGCTCTCTTCAGACTGCCA
Rn Tbp NM_013684.3	CTGGAAGGCCTTGTGTTGAC	GGAGAACAATTCTGGGTTTGA
Hs *PTN* NM_002825.5	AGACTGTCACCATCTCCAAG	GATCCTGTTTGCTGATGTCC
Hs TBP NM_003194.3	AGGTTAGAAGGCCTTGTGCTC	GGGAGGCAAGGGTACATGAG
Mm *Ptn* NM_008973	CAGACCATGAAGACTCAGAG	ACAGTCAGCATTGTGCAGAG
Mm Fgf10 NM_008002	GTTGTTGCCGTCAAAGCCAT	GCCATTGTGCTGCCAGTTAA
Mm Nkx3.1 NM_010921	ACAGTCAGCATTGTGCAGAG	ACCTGAGTGTGAGAGAAGGC
Mm Tbp NM_013684.3	TGCCACACCAGCTTCTGAGA	GCACGAAGTGCAATGGTCTTTAGG

### Northern Blotting Analysis

DNA templates for *Ptn* (rat and human) and *Gapdh* were synthesized by RT-PCR from Rat P0 UGT cDNA or human embryonic prostate cDNA. The DNA template was subcloned into pGEM®-Teasy Vector (Promega, Southampton, UK). The primer sequences were; Rn *Ptn* forward-GCAGTCTGAAGAGAGCTCTG, Rn *Ptn* reverse-CCACTGGCAGAGACAATG and Rn *Gapdh* forward-TTAGCACCCCTGGCCAAGG, Rn *Gapdh* reverse-CTTACTCCTTGGAGGCCATG, Hs *PTN* forward-CAGCTGTGGATACTGCTG, Hs *PTN* reverse-GACAGTCTTCTGGCATTCG. Northern blot hybridization used 5 µg total RNAs as described in Ref. 2. We observed one band (∼1.4 kb) on the Northern blot, corresponding to the predicted size of the Rn *Ptn* transcript. The intensity of the bands was quantified using a phosphoimager, and *Ptn* expression was normalized to *Gapdh*.

### Whole-Mount In Situ Hybridization

The rat *Ptn* template was prepared by TA cloning an 681 bp amplified PCR fragment corresponding to nucleotides 426–1,107 into pGEM®-Teasy. RNA probes were prepared by in vitro transcription using T7 and T3 RNA polymerases (DIG RNA labeling kit, Roche, Burgess Hill, UK). Dissected rat UGT tissue was fixed in 4% paraformaldehyde overnight, dehydrated through graded methanol and stored in 100% methanol at −20°C. RNA in situ hybridization on embryonic and P0 urogenital tracts and cultured ventral prostates were performed using the InsituPro VS robot (Intavis, Bioanalytical Instruments, AG, Cologne, Germany) as described in Ref. 2. Color development times were approximately 8 hr.

### Western Blot Analysis

Protein was isolated from frozen tissue (10–30 organs) using radioimmunoprecipitation assay (RIPA) lysis buffer, and concentration was determined using a Bio-Rad (Hemel Hempstead, UK) protein assay kit. Twenty micrograms of protein extract was run on a Bis–Tris 4–12% gel (Novex pre-cast gels, Invitrogen), and electrotransferred overnight onto nitrocellulose membranes (Immobilon-FL; Millipore, Bedford, MA). The membranes were blocked for 1 hr in 5% milk/TBS, 50 mM Tris–HCl, and 150 mM NaCl and incubated overnight at 4°C with anti-PTN antibody (R&D Systems, Abingdon, UK) diluted 1:100 and anti-β-tubulin antibody (Santa Cruz Biotechnologies, Inc., Santa Cruz, CA) diluted 1:1,000 in 5% milk/TBST (TBS containing 0.05% Tween-20). The membranes were washed in TBST before addition of the appropriate secondary antibody, goat anti-rabbit IRDye 800 (LI-COR Biosciences, Cambridge, UK) and donkey anti-goat Alexa fluor 680 (Invitrogen) diluted 1:10,000 in TBST. Membranes were washed in TBST prior to scanning on the LI-COR (LI-COR Biosciences). Antibody specificity was confirmed by the detection of only one band at the expected size. The intensity of the bands was then quantified, and corrected for loading using β-tubulin.

### Immunohistochemistry

For Ptn, β-catenin, SM α-actin, p63 and BrdU immunostaining, histological sections were pressure cooked in 10 mM citric acid, pH 6.0, for 5 min. Sections to be stained with DAB were treated following a previously published protocol 38. Anti-Ptn antibody (R&D Systems) was diluted 1:200, anti-SM α-actin antibody (Sigma, Poole, UK) was diluted 1:5,000, anti-p63 (Santa Cruz Biotechnologies, Inc.) antibody was diluted 1:300, anti-Pan Cytokeratin was diluted 1:3,000, and anti-BrdU antibody (Fitzgerald Industries International, Inc., Concord, MA) was diluted 1:3,000. Ptn and β-catenin or Ptn and p63 co-localization was performed using anti-Ptn antibody diluted 1:50 and anti β-catenin antibody (Santa Cruz Biotechnologies, Inc.) diluted 1:100 or anti-p63 antibody diluted 1:30. Antibodies were visualized with a species appropriate biotinylated secondary (Vector Laboratories, Burlingame, CA) and avidin–alexafluor 488 or 546 (Molecular Probes, Inc., Eugene, OR). Images were captured using a Provis microscope (Olympus Optical Co., London, UK) equipped with a DCS330 camera (Eastman Kodak Co., Rochester, NY). Confocal microscopy was performed on a Zeiss LSM 510 Laser scanning microscope (Carl Zeiss Microimaging, Inc., Thornwood).

### Organ Culture

Serum-free organ culture was performed as previously described 38. P0 VSUs were placed in culture for 24 hr followed by treatment for 6 and 24 hr ±10^−8^ M testosterone. P0 VPs were cultured for 6 days ±10^−8^ M testosterone and ±3 µg/ml recombinant human Ptn protein (Peprotech, London, UK). Images of organ cultures were captured on a Leica MZ6 dissecting microscope (Leica, Deerfield, IL) with a Leica ICA camera. Organ perimeter and size was measured using NIH image software. Organs were incubated with 100 µg/ml BrdU (Sigma) for 2 hr prior to harvesting.

### Cell Culture

Cells were seeded at 2 × 10^3^ cells/well in 96-well plates (for growth assays) or 6-well plates (for RNA preparation) and grown in DMEM supplemented with 2% or 10% charcoal-stripped fetal calf serum (CSFCS) and 1× penicillin/streptomycin for 24 hr. For RNA, cells were treated ±testosterone (10^−8^ M) for 6 or 24 hr in duplicate. For growth assays, cells were treated with fresh medium ±testosterone ±rhPTN 50 ng/ml–1 µg/ml for 4 days. To measure cell proliferation the Cell Titer 96 AQueous One Solution Cell Proliferation assay was used (Promega UK Ltd., Southampton, UK). The Cell Titer Solution was added directly to the cells in culture medium in a ratio of 1:6 and incubated at 37°C for 100 min followed by absorbance reading at 490 nm.

## RESULTS

### *Ptn* Transcript Expression in the Developing Prostate

**Fig. 1:**
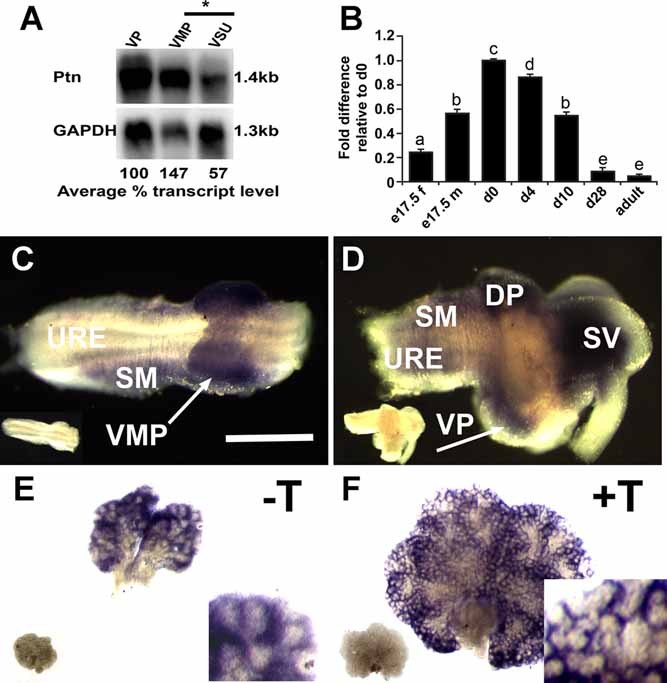
Expression of *Ptn* mRNA in the mesenchyme of male and female UGT. **Panel A**: Comparison of transcript levels in the VMP, VSU and VP (P0) using Northern blot analysis. Transcript abundance was quantified using a phosphoimager and compared with levels expressed in the VP. The VMP exhibited a 2.6-fold enrichment of *Ptn* mRNA compared with the VSU (*P < 0.05) and the VP exhibited a 1.8-fold enrichment of *Ptn* mRNA compared with the VSU. **Panel B**: qRT-PCR analysis of *Ptn* mRNA expression in the male (m) and female (f) UGT at e17.5, and subsequent expression in the VP at P0, P4, P10, P28 and adult prostate. *Ptn* mRNA expression was significantly different in consecutive ages from e17.5 to P28 (^a–e^*P* < 0.001). Mean values were compared between the samples using one-way ANOVA followed by TUKEY multiple comparison. **Panels C–F**: Localization of *Ptn* mRNA using whole-mount in situ hybridization in the male and female UGT and VPs grown in vitro. *Ptn* mRNA expression pattern in female (C) and male (D). The male and female UGT are oriented with the bladder on the right. *Ptn* transcripts were localized to the VMP (C, arrow), and smooth muscle of the female UGT, and the VP (D, arrow), DP, smooth muscle and seminal vesicles of the male UGT. Panels E,F: *Ptn* mRNA distribution in VPs cultured in the absence or presence of testosterone. *Ptn* transcripts were localized to the mesenchyme of VP cultured ±T. The *inset* in the bottom right corner of panel F shows a magnification of VP + T; no antisense probe staining was observed in the epithelial ducts. Specimens hybridized with an antisense probe (purple/blue stain) are shown in each panel, whereas the sense probe is shown as an inset in the bottom left corner of each panel (no signal was observed using the control sense riboprobe). DP, dorsal prostate; URE, urethra; SM, smooth muscle; SV, seminal vesicles. The scale bar represents 200 µm in panels C and D, and 1 mm in panels E and F.

*Ptn* was identified in the rat female VMP by LongSAGE transcriptional profiling with a ratio of 74:63 tags between the VMP/VSU 2. The VSU consists of *V*MP, *s*mooth muscle and *u*rethra and transcripts enriched or specific to the VMP are diluted in the VSU relative to pure VMP. Using Northern blot and qRT-PCR, *Ptn* transcript levels were ∼2.6-fold (Northern) (*P* < 0.05) and 1.5-fold (qRT-PCR, data not shown) higher in the VMP than VSU. The temporal expression of *Ptn* mRNA in the prostate was studied by qRT-PCR (Fig. 1B). *Ptn* was most abundant during the neonatal (P0) and perinatal period (P4, P10) and expression subsequently decreased until adulthood. There was also evidence of higher Ptn levels in males versus females at e17.5 (*P* < 0.001). To examine the spatial distribution of *Ptn* in the male and female UGT, whole-mount in situ hybridization was used to localize *Ptn* transcripts. *Ptn* mRNA was observed in the female in VMP and urethral stroma (Fig. 1C), and in the male in the prostatic mesenchyme (all lobes) and the urethral stroma (Fig. 1D). Additionally, to determine whether *Ptn* mRNA distribution was affected by testosterone, *Ptn* mRNA was localized in male VPs grown in culture ±T (Fig. 1E,F). Organ growth was stimulated by testosterone, and *Ptn* was present in mesenchymal cells throughout the VP, and the distribution of *Ptn* was similar between organs grown with or without testosterone. There was no evidence of epithelial *Ptn* expression (insets, Fig. 1E,F) though our studies do not rule out low level epithelial expression or expression within epithelial subsets.

### Ptn Protein Localization in the Male and Female Urethra and Prostate

**Fig. 2:**
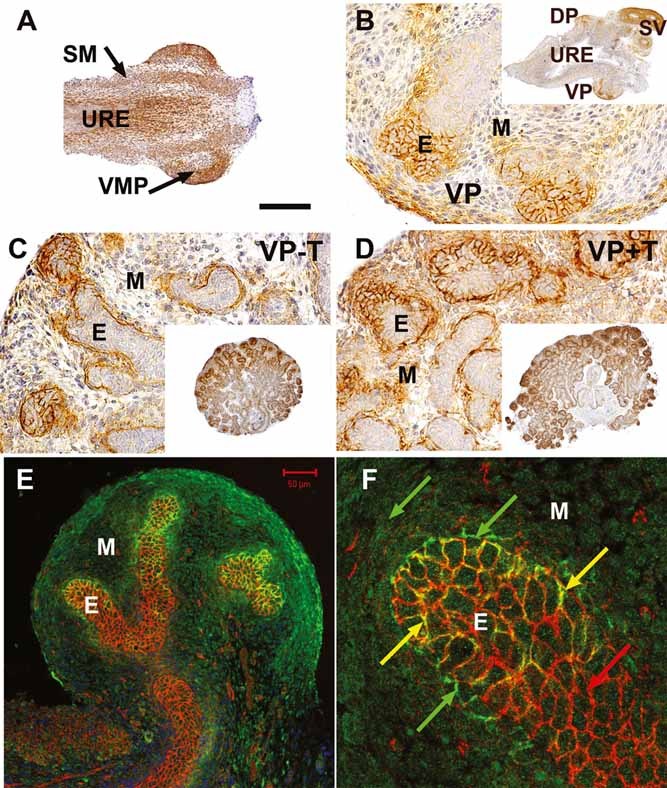
Localization of Ptn in the male and female UGT. The distribution of Ptn protein in the male and male and female UGT (P0) and VP organs grown in vitro was examined by immunohistochemistry. The UGT sections are orientated so that the bladder is on the right-hand side. **Panel A**: Ptn was expressed in the VMP, periurethral mesenchyme and smooth muscle of the female UGT (marked by arrows). **Panel B**: Ptn was present in the mesenchyme (M) of the VP in the male. Ptn expression was also strongly associated with the epithelial cells (E) at the tips of the ducts of the VP. The *inset* in the top right corner shows the whole male UGT with Ptn expression in the DP and SV. **Panels C**,**D**: VPs cultured ±T exhibited mesenchymal expression of Ptn and also association of Ptn with surface of ductal epithelia at the tips of the cords. The *inset* in the bottom right corner shows the whole VP organ. Testosterone did not alter Ptn distribution. **Panels E**,**F**: Ptn and β-catenin were co-localized in the male VP, using immunofluorescence; Ptn was stained green, β-catenin red, and co-localization of Ptn and β-catenin proteins appears yellow. Panel E: Ptn and β-catenin were co-localized to the epithelial cell surface at the tips of the prostatic ducts (yellow). Panel F: A magnified area of the VP in panel E, showing an epithelial duct tip. Ptn protein expression was not co-localized with β-catenin in the mesenchyme or most of the epithelial duct basement membrane (green arrows), and was absent from other areas of the epithelial surface (red arrows), but was co-localized in some sub-regions (yellow arrows). The scale bars shown are 200 µm in panel A, 50 µm in panels B–D, 50 µm in panel E and 15 µm in panel F.

Ptn protein was localized in the male and female urethra and prostate by immunohistochemistry, and showed a similar pattern to the in situ hybridization data. Ptn was observed in the VMP, periurethral mesenchyme and smooth muscle layer of the female (Fig. 2A, arrows), and the mesenchyme, periurethral mesenchyme, and smooth muscle of the prostate (Fig. 2B). The strongest mesenchymal Ptn staining in the VP was at the periphery. Ptn was also localized to the ductal epithelia of the VP, where a gradient of Ptn expression was observed; Ptn was present at the tips of the ducts and was absent proximal to the urethra. Additionally, Ptn expression was examined in VP organs cultured ±T (Fig. 2C,D) and Ptn distribution was similar to the VP in vivo (Fig. 2B); androgens did not alter Ptn distribution. To examine the precise localization of Ptn in VP epithelia, we co-localized Ptn with β-catenin, a cell surface marker (Fig. 2E,F). Ptn was expressed in the mesenchyme of the VP, and Ptn and β-catenin were co-expressed at the cell surface of ductal epithelial cells. The gradient of Ptn expression was confirmed with strong Ptn staining at the ductal tips and less staining proximal to the urethra. Additionally, co-localization of Ptn with p63 showed that Ptn was associated with both p63 positive and p63 negative cells (Supplementary Fig. 1). Ptn distribution was partially inverse to the pattern associated with smooth muscle differentiation around epithelial ducts. We propose that, since *Ptn* mRNA was not observed in epithelia, Ptn at the epithelial surface was derived from the mesenchyme.

### Ptn Is Involved in Prostatic Growth and Branching

**Fig. 3:**
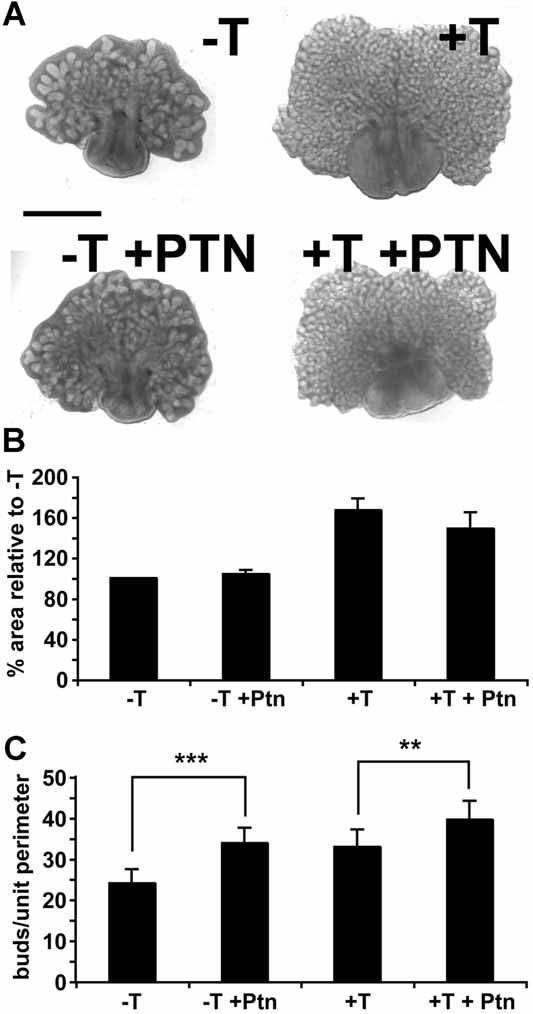
The effect of recombinant hPTN on VPs grown in vitro. P0 VPs were cultured in the presence or absence of testosterone (10^−8^ M) and/or hPTN (3 µg/ml). **Panel A**: Whole-mount images of male VP organ cultures after 6 days; addition of hPTN led to small effects upon organ size and morphology. Scale bar represents 1 mm. **Panel B**: Graph showing the mean two-dimensional area of VPs relative to −T, ±SEM. Addition of hPTN led to a modest reduction in the size of organs grown in the presence of T, but not in organs grown in the absence of T. **Panel C**: Graph showing the mean number of epithelial bud tips around the periphery of cultured VPs, expressed as a ratio of organ perimeter (mean number of buds per 1,000 pixels perimeter ±SEM). Addition of hPTN led to a statistically significant (Student's *t*-test, **P < 0.01 and ***P < 0.001) increase in the number of epithelial bud tips/unit perimeter in VPs grown ±T.

The role of Ptn in prostate growth and morphogenesis was investigated using cultures of VP organs grown in vitro and treated with testosterone and recombinant hPTN protein (Fig. 3A). Addition of testosterone led to an increase in organ size and branching; however, addition of hPTN had relatively modest effects either in the presence or absence of testosterone (n = 5 experiments, 45 organs). The two dimensional area of VP organs grown ±T, ±3 µg/ml hPTN was measured using NIH imaging software (Fig. 3B, and Supplementary Table A). Addition of hPTN to VP organs had no statistically significant effect upon organ area, in the presence (*P* = 0.11) or absence (*P* = 0.4) of testosterone. hPTN (Peprotech) was added to organ cultures at physiological levels (∼0.94 µg/ml tissue volume) as used previously, to determine a functional response in vitro 39. To investigate a role for hPTN in branching morphogenesis, the number of epithelial bud tips around the periphery of VPs cultured ±T and ±hPTN were counted. The number of tips was expressed as a ratio to organ perimeter (tips per 1,000 pixels perimeter) to control for changes in organ size (Fig. 3C, and values are listed in Supplementary Table B). Treatment of VPs with hPTN caused an increase in the number of tips/1,000 pixels perimeter in the presence (*P* < 0.01) or absence (*P* < 0.001) of testosterone, and we suggest that Ptn has a role in increasing branching morphogenesis in the prostate.

### Ptn Effects Upon Cellular Proliferation and Differentiation

**Fig. 4:**
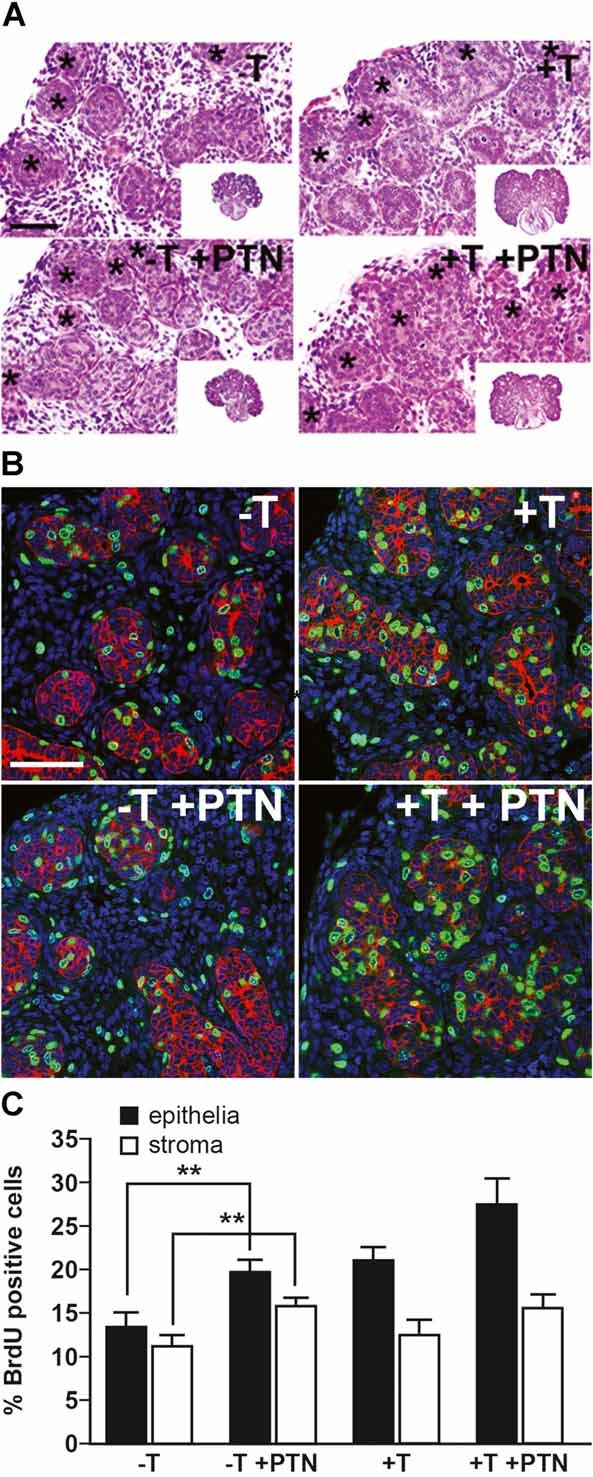
The effect of hPTN on cellular proliferation in the proximal and distal regions of the prostate. Rat VPs (P0) were grown ±T and ±hPTN for 6 days and BrdU was added to the culture medium for 2 hr prior to harvesting. **Panel A**: Histology (hematoxylin and eosin staining) of cultured VPs after 6 days, asterisks (*) mark epithelial buds at the periphery of the organ. Scale bar represents 100 µm. **Panel B**: Immunohistochemistry of VP epithelial ducts distal to the urethra under different treatment conditions showing BrdU incorporation (green) in the epithelial cells (red); nuclei were stained with Topro (blue). Scale bar represents 100 µm. **Panel C**: Graphs of BrdU incorporation ±SEM illustrating the rates of proliferation of stroma and epithelia. Addition of hPTN led to an increase in stromal and epithelial cell proliferation only in the absence of testosterone (Student's *t*-test, **P < 0.01).

To examine the effect of hPTN upon the cellular differentiation and proliferation of VPs grown in vitro (n = 3, 27 organs) we examined the organs by histology and calculated proliferative rates using immunohistochemistry for BrdU incorporation and stromal and epithelial markers (Fig. 4). Histology of the VP organs showed no substantial changes in organ morphology or stromal and epithelial distribution (Fig. 4A). Analysis of stromal and epithelial differentiation using SM α-actin and p63 showed no difference in cellular differentiation after treatment with hPTN, but confirmed increased branching of ductal tips at the periphery of the VP organs (Supplementary Fig. 2). The proliferative index of the epithelial and mesenchymal compartments in the peripheral region (distal to the urethra) was calculated for VPs cultured ±T, ±hPTN (Fig. 4B,C and values are listed in Supplementary Tables C and D). hPTN significantly increased epithelial (*P* < 0.05) and stromal (*P* < 0.01) proliferation in the absence of testosterone.

### PTN Increases Proliferation of Embryonic and Cancer-Associated Fibroblasts, and BPH1 Cells

**Fig. 5:**
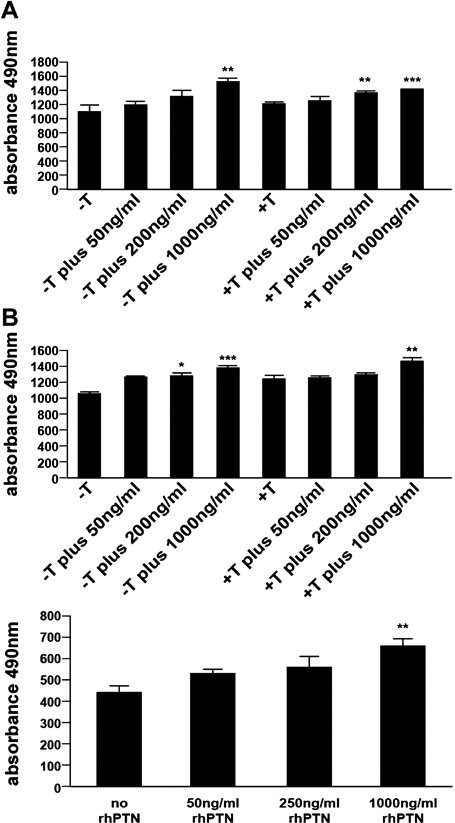
The effect of hPTN on cultures of human prostate cancer fibroblasts and BPH1 cells. **Panel A**: Human embryonic prostate fibroblasts (EPFs) isolated from 15 to 18 weeks male UGT were grown ± testosterone and with increasing amounts of hPTN (50–1,000 ng/ml). **Panel B**: Human prostate CAFs isolated from adult male TURP. **Panel C**: BPH1 cells. The proliferation of the cells was measured by MTT assay and the values at day 4 are presented and the measurement at 490 nm is relative to the cell number. Addition of hPTN (50 ng/ml–1 µg/ml) led to a significant increase in the cell number of EPFs, CAFs and BPH1 cells after 4 days both in the presence or absence of testosterone. One-way ANOVA with TUKEY multiple comparison; *P < 0.05, **P < 0.01, ***P < 0.001.

The effect of hPTN upon embryonic human prostate fibroblasts and adult CAFs was measured by proliferation assay (MTT) (Fig. 5A and Supplementary Table E). Addition of hPTN (1 µg/ml) in the absence of testosterone and hPTN (200 ng/ml–1 µg/ml) in the presence of testosterone, led to a statistically significant (*P* < 0.01, one-way ANOVA) increase in the number of embryonic human prostate fibroblasts. We next examined the effect of Ptn upon the proliferation of adult prostate CAFs. Average values for cancer associated fibroblasts, derived from eight patients are shown in Figure 5B (and Supplementary Table F). Addition of recombinant hPTN protein (200 ng/ml–1 µg/ml) in the absence of testosterone or 1 µg/ml hPTN in the presence of testosterone led to a statistically significant increase in CAF cell number (*P* < 0.01) (one-way ANOVA). To investigate the effect of hPTN on epithelial cells we used BPH1 cells (−T, ±hPTN 50 ng/ml–1 µg/ml) (Fig. 5C and Supplementary Table G). BPH1 cells were used as a model of non-tumorigenic human prostate epithelia which do not express a functional androgen receptor, and thus testosterone was not added to the treatment groups. Addition of hPTN (1 µg/ml) to BPH1 cells led to a statistically significant increase in cell number (*P* < 0.01) (one-way ANOVA). Taken together, Ptn protein stimulated the growth of human embryonic fibroblasts, CAFs and BPH1 cells in the presence or absence of testosterone.

### Differential Expression of *Ptn* mRNA Between Normal and Cancer-Associated Fibroblasts, and Males and Females

**Fig. 6:**
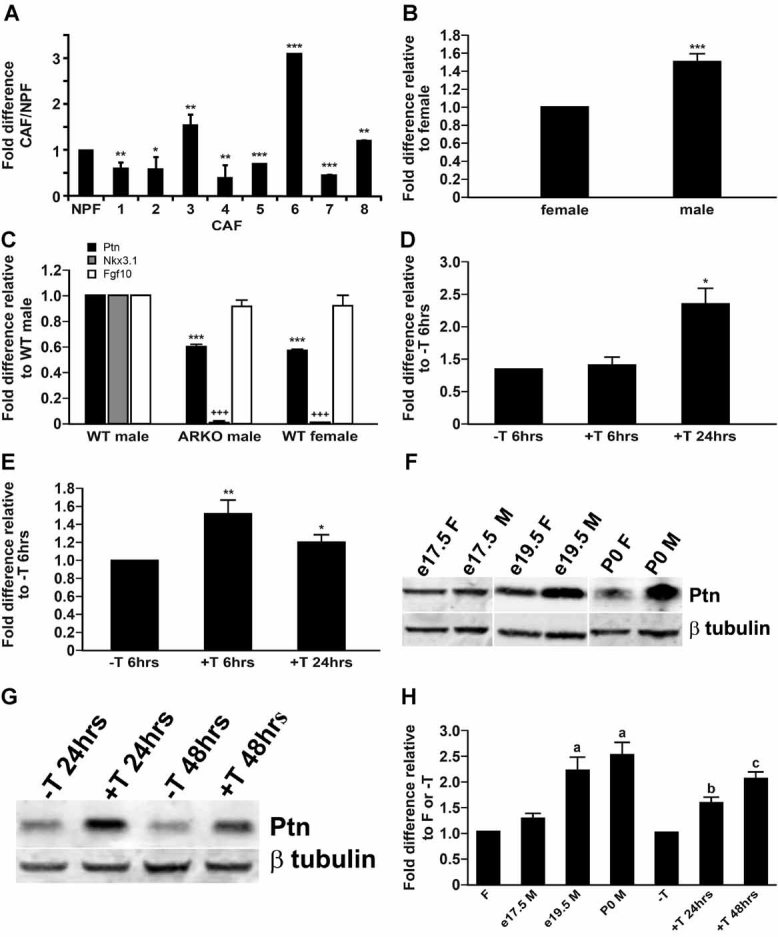
Expression of *PTN* mRNA in CAFs versus NPFs, male versus female urethra, and regulation by androgens. **Panel A**: The expression of *PTN* mRNA showed a decrease in CAFs compared to NPFs in five of eight CAF/NPF pairs, when quantified by qRT-PCR. **Panel B**: qRT-PCR analysis of *PTN* mRNA expression in human embryonic male and female urethra/UGT (15–18 weeks). The embryonic male UGT is exposed to significantly higher levels of circulating testosterone, produced by the fetal testes, than the embryonic female UGT 48. *PTN* mRNA levels were ∼1.5-fold higher in the embryonic male versus female UGT. **Panel C**: Quantitative PCR for *Ptn* mRNA in the UGT of male mice, ARKO male mice and female mice. ARKO mice lack AR and are a model for androgen action; they develop testes but lack secondary sex accessory tissues. *Ptn* mRNA was less abundant in ARKO males and wt females compared to wt males. NKx3.1, an epithelial marker of prostate identity which is known to be regulated by androgens, was included as a control and showed a marked reduction in level between wt males and ARKOs males or wt females. Fgf10, a ligand expressed in the stroma, showed no change between wt males, ARKO males, or females, suggesting that it is not androgen-regulated and that the mesenchymal composition between these samples is similar. **Panel D**: qRT-PCR analysis of rat VSUs grown ± testosterone for 6 and 24 hr; testosterone increased *Ptn* mRNA levels by twofold at 24 hr but not at 6 hr. **Panel E**: qRT-PCR for *PTN* mRNA in human embryonic prostate fibroblasts cultured in the absence of testosterone followed by 6 and 24 hr treatment with testosterone. *PTN* transcript expression increased in the presence of testosterone at 6 hr and was partially elevated at 24 hr. **Panels F**,**G**: Western blot analysis of Ptn (19 kDa) and β-tubulin (50 kDa); male and female rat UGT at e17.5, e19.5 and P0 (F), and VSU organs grown in the presence or absence of testosterone for 24 and 48 hr (G). β-tubulin was used as the loading control and for normalization. **Panel H**: Quantification of Western blot analysis in panels F and G, showing fold difference in Ptn expression in VSU organs grown −T versus +T 24 and 48 hr; testosterone increased Ptn levels at 24 and 48 hr and a difference in Ptn expression between female versus male rat UGT (e17.5, 19.05, and P0). Panels A,B,D,E, Student's *t*-test; *P < 0.05, **P < 0.01, ***P < 0.001. Panels C,H: One-way ANOVA with TUKEY multiple comparison; (C) ^***,+++^*P* < 0.001, (H) ^b–d^*P* < 0.001.

To examine if *Ptn* mRNA was differentially expressed between CAFs and NPFs, we compared *Ptn* transcript levels in eight pairs of functionally tested patient-matched NPF and CAF samples 7. qRT-PCR analysis (Fig. 6A) determined that there was between 1.6- and 2.5-fold (*P* < 0.05–0.001) less *Ptn* expression in CAFs compared to NPFs in 5 of 8 samples. In samples 3 and 6, *Ptn* increased in CAFs versus NPFs and this indicates the heterogeneity inherent in patient CAF samples. CXCL12 was found to be increased in six of eight of the CAF samples, similar to findings in breast cancer stroma (data not shown) 40,41. Next, we investigated whether *Ptn* exhibited sexually dimorphic expression in the developing human urethra and prostate. *Ptn* transcript expression was compared between the male and female human urethra (15–18 weeks) by qRT-PCR and Northern blot. qRT-PCR analysis of *PTN* mRNA expression demonstrated *PTN* transcript levels were 1.5-fold higher in the male compared to female (*P* < 0.001) (Fig. 6B). Northern blot analysis determined *PTN* transcript expression was ∼2-fold higher in the male compared to female (data not shown). *Ptn* mRNA (*P* < 0.001, Fig. 1B), and protein (1.3-fold, Fig. 6H) expression was also higher in e17.5 male versus female rat UGT, where there is little difference in tissue morphology. We have shown sexually dimorphic expression of *Ptn* in the developing UGT and as Ptn is expressed in the mesenchyme only of the rat and human prostate 15, it is not due to the presence of prostatic ducts in the male and their absence in female.

### Ptn Expression Is Increased by Androgens and Androgen Receptor

To determine whether androgens were involved in the regulation of *Ptn* mRNA expression we examined its expression in feminized ARKO male mice that lack AR and which cannot respond to androgens. *Ptn* transcripts were measured by qRT-PCR in the UGT of ARKO males, wt males, and wt females (Fig. 6C). As controls, we included Nkx3.1 a gene expressed in epithelia and known to be androgen regulated, as well as Fgf10, a mesenchymally expressed gene unlikely to be regulated by androgens. Nkx3.1 mRNA levels, were substantially higher in wt males compared to ARKO males and females (*P* < 0.001, one-way ANOVA) consistent with its epithelial expression, while Fgf10 mRNA levels were similar in all three groups. *Ptn* mRNA was 1.6-fold lower in ARKO males and wt females (*P* < 0.001, one-way ANOVA), compared to wt males, suggesting that androgens and AR increase *Ptn* transcript levels (Fig. 6C). Next, we examined whether *Ptn* mRNA levels were affected by testosterone using female VMP organ rudiments grown in vitro (±T). Female VMP was cultured for 24 hr followed by 6 or 24 hr of treatment with testosterone (Fig. 6D). VMPs (n = 4, 40 organs) and not VPs were used for this experiment to minimize the potential carry over of residual testosterone in male VPs. No changes in gross tissue morphology of the stroma or epithelia were observed during treatment of VMPs for 6–24 hr in vitro. The results, expressed as fold difference in *Ptn* mRNA expression relative to −T were as follows: −T = 1 versus +T 6 hr = 1.07 ± 0.03 (Student's *t*-test, *P* = 0.3), −T versus +T 24 hr = 2.22 ± 0.29 (Student's *t*-test, *P* = 0.03). *Ptn* transcript levels were upregulated by testosterone in cultures of rat female VMP. We next examined the effects of testosterone upon *PTN* mRNA levels in primary human embryonic prostate fibroblast cells (n = 3), treated ±T for 6 and 24 hr. The fold difference in *PTN* mRNA levels were: −T = 1 versus +T 6 hr = 1.52 ± 0.09 (Student's *t*-test, *P* = 0.0082) and −T versus +T 24 hr = 1.20 ± 0.08 (Student's *t*-test, *P* = 0.046) (Fig. 6E). To determine if *PTN* mRNA expression in CAFs was androgen-regulated, *PTN* mRNA was measured in CAFs grown in the presence or absence of testosterone for 6 or 24 hr (n = 3) by qRT-PCR and Northern blotting (Supplementary Fig. 3). *PTN* transcript levels in CAFs showed very small changes in response to testosterone at any of the time points. We determined AR mRNA and protein expression in CAFs by RT-PCR and immunohistochemistry (data not shown), prior to performing the experiments. We note that throughout our studies Ptn transcript levels were detectable in females or the absence of testosterone, and that androgens and AR increased these levels. This suggests that there is a component of Ptn expression which is androgen independent.

To determine whether Ptn protein expression was regulated by androgens, we examined sexually dimorphic expression of Ptn and its response to androgens in vitro. We examined Ptn protein expression in developing male and female rat prostate/urethra (e17.5, e19.5, and P0) using Western blot analysis, and observed sexually dimorphic expression (Fig. 6F). Also, we measured Ptn in VSU organs grown ±T for 24 or 48 hr using Western blot analysis (Fig. 6G) (n = 3, 38 organs). Quantification of Ptn protein in VSU organs grown in the presence of testosterone was expressed as fold difference relative the absence of testosterone at each time point. The data were as follows: −T versus +T 24 hr = 1.6 ± 0.1 (Student's *t*-test, *P* = 0.002), −T versus +T 48 hr 2.1 ± 0.1 (Student's *t*-test, *P* = 0.0003) (Fig. 6H). The data demonstrated that Ptn protein was upregulated by testosterone in cultures of rat female VMP. Quantification of Ptn protein in the prostate was expressed as fold difference relative to the female urethra at each time point. The results were as follows: e17.5 female versus e17.5 male = 1.3 ± 0.1 (Student's *t*-test, *P* = 0.057), e19.5 female versus e19.5 male = 2.2 ± 0.3 (Student's *t*-test, *P* = 0.014), P0 female versus P0 male = 2.5 ± 0.2 (Student's *t*-test, *P* = 0.007) (Fig. 6H). Ptn protein levels showed a sexually dimorphic expression, with higher Ptn expression in the male versus female.

## DISCUSSION

We propose that Ptn signaling is involved in regulation of growth and branching morphogenesis in the developing prostate, and that *Ptn* expression is increased by androgens. We identified *Ptn* in a gene profiling study of VMP mesenchyme 2 which is a specialized area of mesenchyme that contains prostate inductive activity 42. Given the importance of VMP mesenchyme in regulating prostate organogenesis, the identification of molecules that can mediate its effects is important and we propose that Ptn is one of many paracrine regulators made in VMP mesenchyme that regulate prostate growth. *Ptn* expression is associated with the development of organs that are regulated by epithelial–mesenchymal interactions and has also been described as a proto-oncogene. *Ptn* expression in the mesenchyme plays a role in the developing kidney, lung, and mammary gland 25,26,43, and has been localized to the stroma of adult prostate, and prostate cancer 15. The role of Ptn in organogenesis has been difficult to examine given co-expression and redundancy of midkine; however, mice doubly null for Ptn and midkine show female infertility and reproductive abnormalities but no effects in males have been reported 28. Were Ptn an essential mediator of androgen action, loss of Ptn might be expected to show impairment of masculinization similar to that seen in Tfm or ARKO mice, and such molecules remain to be identified.

We suggest that Ptn is a factor that regulates the proliferation of human and rodent prostatic stromal and epithelial cells in developmental and disease models. Given that transcripts for *Ptn* are stromal, it suggests that Ptn functions as an autocrine regulator of stroma and a paracrine regulator of epithelia. The paracrine function of Ptn is supported by the localization of Ptn on the surface of epithelial cells in growing ductal tips, where Ptn protein was abundant, in contrast to its absence from ducts proximal to the urethra. It will be important to establish the expression patterns of the three receptors that bind Ptn and transduce its signal, as this will define Ptn responsive cells within the stromal and epithelial compartments, and these may include key subsets of cells such as progenitors. Co-localization of Ptn with β-catenin on the surface of epithelial cells is notable since β-catenin is a substrate of receptor protein tyrosine phosphatase RPTB/ξ 16, a well-studied Ptn receptor. Ptn is known to interact with the extracellular matrix of undifferentiated cells 23 and Ptn distribution patterns similar to our observations have been reported in the mouse genital tubercle, where Ptn is expressed in the mesenchyme and on the surface of epithelial cells 23. Ptn is also expressed in the metanephric mesenchyme and on the basement membrane of the uteric bud at the onset of kidney development 26. A gradient of Ptn is present in the uteric bud, similar to our findings in VP epithelia. Sakurai et al suggested Ptn may be acting as “a classic morphogen” of the mesenchyme that induced phenotypic changes in epithelia according to its concentration. We observed that Ptn had a small effect on branching morphogenesis at the periphery of the developing prostate in vitro, and established that it did not lead to mis-differentiation of stroma or epithelia when examined by immunohistochemistry for p63 or SMA (Supplementary Fig. 2). Furthermore, addition of hPTN to VPs in culture led to a significant increase in the number of bud tips and an increase in the stromal and epithelial cell proliferative rate at the periphery of the organs in the absence of testosterone. We propose that Ptn functions as a classic morphogen in the developing prostate, since it is made by inductive mesenchyme of the VMP and shows similar activities in other organs such as kidney and genital tubercle 23,26.

Ptn has been shown to be mitogenic for a variety of fibroblast, epithelial and endothelial cell lines, including normal human prostate (PNT1A) and prostate cancer epithelial lines (DU145, PC3, and LNCaP) 15,30. We examined the effect of hPTN on primary human prostate embryonic fibroblasts, cancer associated fibroblasts and BPH1 cells in vitro, and found that addition of hPTN led to a significant increase in cell proliferation. *Ptn* mRNA and protein are expressed in the stromal cells of normal adult prostate, prostate cancer and benign prostatic hyperplasia (BPH), and Ptn protein is also expressed in prostate tumor epithelia 15. Surprisingly, when we examined *PTN* mRNA expression levels in matched pairs of CAFs/NPFs, five out of eight samples showed lower levels of *PTN* transcripts in CAFs compared to NPFs, and a recent study has confirmed a reduction of *PTN* in reactive prostate cancer stroma 44. Breast cancer studies have reported conflicting data on *PTN* mRNA regulation in normal and cancer cells, reviewed in Ref. 36. Different approaches to attenuate Ptn function in tumor cells were found to decrease tumor growth in vivo, thus constitutive Ptn signaling was a rate-limiting factor in the pathogenesis of these tumors.

In the developing prostate and normal adult prostate the effects of androgens upon epithelial cells are mediated via AR that is expressed in the mesenchyme or stroma, respectively 45,46. However, there is little knowledge of the genes and pathways that mediate the effects of androgens on normal prostate growth or exactly how the paracrine effects of androgens are mediated. *PTN* mRNA exhibited a sexually dimorphic pattern of expression in the human fetal prostate/urethra; *PTN* transcript levels were higher in the male, with elevated androgen levels, compared to the female. Similarly there were lower levels of *Ptn* mRNA in the urethra of female mice and ARKO males (that lack AR) when compared with wt male mice. Ptn protein also showed a sexually dimorphic difference between male and female rat urethra/prostate. Both Ptn mRNA and protein were upregulated by testosterone in organ cultures of the female VMP. We have shown that androgens increase Ptn levels in several different systems; however, Ptn expression is evident in females or the absence of androgens, which indicates there is an androgen independent element of Ptn expression. Gene profiling studies have confirmed the correlation between androgen levels and *Ptn* transcript levels in the prostate 47. Such correlative data do not provide direct evidence for Ptn function as an “andromedin,” which would require direct experimental proof. Since prostate cancer is sensitive to androgens, we examined whether *Ptn* mRNA was affected by exposure to testosterone in three CAF isolates. Surprisingly, we found that T did not significantly alter *Ptn* mRNA levels in CAFs (Supplementary Fig. 3). There are several possible explanations for this, however, we suggest that it may reflect dysregulation of androgen signaling in CAFs and contributes to their pro-tumorigenic signaling.

## CONCLUSION

Our data suggest that in the prostate Ptn functions as a regulator of both mesenchymal and epithelial proliferation, and that androgens regulate Ptn levels.

## References

[1] Marker PC, Donjacour AA, Dahiya R, Cunha GR (2003). Hormonal, cellular, and molecular control of prostatic development. Dev Biol.

[2] Vanpoucke G, Orr B, Grace OC, Chan R, Ashley GR, Williams K, Franco OE, Hayward SW, Thomson AA (2007). Transcriptional profiling of inductive mesenchyme to identify molecules involved in prostate development and disease. Genome Biol.

[3] Zhang TJ, Hoffman BG, Ruiz de Algara T, Helgason CD (2006). SAGE reveals expression of Wnt signalling pathway members during mouse prostate development. Gene Expr Patterns.

[4] Bhowmick NA, Neilson EG, Moses HL (2004). Stromal fibroblasts in cancer initiation and progression. Nature.

[5] Cunha GR, Hayward SW, Wang YZ (2002). Role of stroma in carcinogenesis of the prostate. Differentiation.

[6] Barclay WW, Woodruff RD, Hall MC, Cramer SD (2005). A system for studying epithelial–stromal interactions reveals distinct inductive abilities of stromal cells from benign prostatic hyperplasia and prostate cancer. Endocrinology.

[7] Olumi AF, Grossfeld GD, Hayward SW, Carroll PR, Tlsty TD, Cunha GR (1999). Carcinoma-associated fibroblasts direct tumor progression of initiated human prostatic epithelium. Cancer Res.

[8] Tuxhorn JA, McAlhany SJ, Dang TD, Ayala GE, Rowley DR (2002). Stromal cells promote angiogenesis and growth of human prostate tumors in a differential reactive stroma (DRS) xenograft model. Cancer Res.

[9] Thomson AA, Cunha GR (1999). Prostatic growth and development are regulated by FGF10. Development.

[10] Lamm ML, Podlasek CA, Barnett DH, Lee J, Clemens JQ, Hebner CM, Bushman W (2001). Mesenchymal factor bone morphogenetic protein 4 restricts ductal budding and branching morphogenesis in the developing prostate. Dev Biol.

[11] Orr B, Grace OC, Vanpoucke G, Ashley GR, Thomson AA (2009). A role for notch signaling in stromal survival and differentiation during prostate development. Endocrinology.

[12] Prins GS, Putz O (2008). Molecular signaling pathways that regulate prostate gland development. Differentiation.

[13] Thomson AA (2008). Mesenchymal mechanisms in prostate organogenesis. Differentiation.

[14] Nakanishi T, Kadomatsu K, Okamoto T, Tomoda Y, Muramatsu T (1997). Expression of midkine and pleiotropin in ovarian tumors. Obstet Gynecol.

[15] Vacherot F, Caruelle D, Chopin D, Gil-Diez S, Barritault D, Caruelle JP, Courty J (1999). Involvement of heparin affin regulatory peptide in human prostate cancer. Prostate.

[16] Maeda N, Nishiwaki T, Shintani T, Hamanaka H, Noda M (1996). 6B4 proteoglycan/phosphacan, an extracellular variant of receptor-like protein-tyrosine phosphatase zeta/RPTPbeta, binds pleiotrophin/heparin-binding growth-associated molecule (HB-GAM). J Biol Chem.

[17] Raulo E, Chernousov MA, Carey DJ, Nolo R, Rauvala H (1994). Isolation of a neuronal cell surface receptor of heparin binding growth-associated molecule (HB-GAM). Identification as N-syndecan (syndecan-3). J Biol Chem.

[18] Stoica GE, Kuo A, Aigner A, Sunitha I, Souttou B, Malerczyk C, Caughey DJ, Wen D, Karavanov A, Riegel AT, Wellstein A (2001). Identification of anaplastic lymphoma kinase as a receptor for the growth factor pleiotrophin. J Biol Chem.

[19] Zhang L, Rees MC, Bicknell R (1995). The isolation and long-term culture of normal human endometrial epithelium and stroma. Expression of mRNAs for angiogenic polypeptides basally and on oestrogen and progesterone challenges. J Cell Sci.

[20] Li YS, Milner PG, Chauhan AK, Watson MA, Hoffman RM, Kodner CM, Milbrandt J, Deuel TF (1990). Cloning and expression of a developmentally regulated protein that induces mitogenic and neurite outgrowth activity. Science.

[21] Rauvala H, Vanhala A, Castren E, Nolo R, Raulo E, Merenmies J, Panula P (1994). Expression of HB-GAM (heparin-binding growth-associated molecules) in the pathways of developing axonal processes in vivo and neurite outgrowth in vitro induced by HB-GAM. Brain Res Dev Brain Res.

[22] Ledoux D, Caruelle D, Sabourin JC, Liu J, Crepin M, Barritault D, Courty J (1997). Cellular distribution of the angiogenic factor heparin affin regulatory peptide (HARP) mRNA and protein in the human mammary gland. J Histochem Cytochem.

[23] Mitsiadis TA, Salmivirta M, Muramatsu T, Muramatsu H, Rauvala H, Lehtonen E, Jalkanen M, Thesleff I (1995). Expression of the heparin-binding cytokines, midkine (MK) and HB-GAM (pleiotrophin) is associated with epithelial-mesenchymal interactions during fetal development and organogenesis. Development.

[24] Vanderwinden JM, Mailleux P, Schiffmann SN, Vanderhaeghen JJ (1992). Cellular distribution of the new growth factor pleiotrophin (HB-GAM) mRNA in developing and adult rat tissues. Anat Embryol (Berl).

[25] Bernard-Pierrot I, Delbe J, Heroult M, Rosty C, Soulie P, Barritault D, Milhiet PE, Courty J (2004). Heparin affin regulatory peptide in milk: Its involvement in mammary gland homeostasis. Biochem Biophys Res Commun.

[26] Sakurai H, Bush KT, Nigam SK (2001). Identification of pleiotrophin as a mesenchymal factor involved in ureteric bud branching morphogenesis. Development.

[27] Amet LE, Lauri SE, Hienola A, Croll SD, Lu Y, Levorse JM, Prabhakaran B, Taira T, Rauvala H, Vogt TF (2001). Enhanced hippocampal long-term potentiation in mice lacking heparin-binding growth-associated molecule. Mol Cell Neurosci.

[28] Muramatsu H, Zou P, Kurosawa N, Ichihara-Tanaka K, Maruyama K, Inoh K, Sakai T, Chen L, Sato M, Muramatsu T (2006). Female infertility in mice deficient in midkine and pleiotrophin, which form a distinct family of growth factors. Genes Cells.

[29] Bowden ET, Stoica GE, Wellstein A (2002). Anti-apoptotic signaling of pleiotrophin through its receptor, anaplastic lymphoma kinase. J Biol Chem.

[30] Fang W, Hartmann N, Chow DT, Riegel AT, Wellstein A (1992). Pleiotrophin stimulates fibroblasts and endothelial and epithelial cells and is expressed in human cancer. J Biol Chem.

[31] Yeh HJ, He YY, Xu J, Hsu CY, Deuel TF (1998). Upregulation of pleiotrophin gene expression in developing microvasculature, macrophages, and astrocytes after acute ischemic brain injury. J Neurosci.

[32] Choudhuri R, Zhang HT, Donnini S, Ziche M, Bicknell R (1997). An angiogenic role for the neurokines midkine and pleiotrophin in tumorigenesis. Cancer Res.

[33] Christman KL, Fang Q, Kim AJ, Sievers RE, Fok HH, Candia AF, Colley KJ, Herradon G, Ezquerra L, Deuel TF, Lee RJ (2005). Pleiotrophin induces formation of functional neovasculature in vivo. Biochem Biophys Res Commun.

[34] Czubayko F, Schulte AM, Berchem GJ, Wellstein A (1996). Melanoma angiogenesis and metastasis modulated by ribozyme targeting of the secreted growth factor pleiotrophin. Proc Natl Acad Sci USA.

[35] Sugino T, Kusakabe T, Hoshi N, Yamaguchi T, Kawaguchi T, Goodison S, Sekimata M, Homma Y, Suzuki T (2002). An invasion-independent pathway of blood-borne metastasis: A new murine mammary tumor model. Am J Pathol.

[36] Perez-Pinera P, Chang Y, Deuel TF (2007). Pleiotrophin, a multifunctional tumor promoter through induction of tumor angiogenesis, remodeling of the tumor microenvironment, and activation of stromal fibroblasts. Cell Cycle.

[37] De Gendt K, Atanassova N, Tan KA, de Franca LR, Parreira GG, McKinnell C, Sharpe RM, Saunders PT, Mason JI, Hartung S, Ivell R, Denolet E, Verhoeven G (2005). Development and function of the adult generation of Leydig cells in mice with Sertoli cell-selective or total ablation of the androgen receptor. Endocrinology.

[38] Thomson AA, Timms BG, Barton L, Cunha GR, Grace OC (2002). The role of smooth muscle in regulating prostatic induction. Development.

[39] Li J, Wei H, Chesley A, Moon C, Krawczyk M, Volkova M, Ziman B, Margulies KB, Talan M, Crow MT, Boheler KR (2007). The pro-angiogenic cytokine pleiotrophin potentiates cardiomyocyte apoptosis through inhibition of endogenous AKT/PKB activity. J Biol Chem.

[40] Allinen M, Beroukhim R, Cai L, Brennan C, Lahti-Domenici J, Huang H, Porter D, Hu M, Chin L, Richardson A, Schnitt S, Sellers WR, Polyak K (2004). Molecular characterization of the tumor microenvironment in breast cancer. Cancer Cell.

[41] Orimo A, Gupta PB, Sgroi DC, Arenzana-Seisdedos F, Delaunay T, Naeem R, Carey VJ, Richardson AL, Weinberg RA (2005). Stromal fibroblasts present in invasive human breast carcinomas promote tumor growth and angiogenesis through elevated SDF-1/CXCL12 secretion. Cell.

[42] Timms BG, Lee CW, Aumuller G, Seitz J (1995). Instructive induction of prostate growth and differentiation by a defined urogenital sinus mesenchyme. Microsc Res Tech.

[43] Lu J, Qian J, Izvolsky KI, Cardoso WV (2004). Global analysis of genes differentially expressed in branching and non-branching regions of the mouse embryonic lung. Dev Biol.

[44] Dakhova O, Ozen M, Creighton CJ, Li R, Ayala G, Rowley D, Ittmann M (2009). Global gene expression analysis of reactive stroma in prostate cancer. Clin Cancer Res.

[45] Cunha GR, Chung LW (1981). Stromal–epithelial interactions—I. Induction of prostatic phenotype in urothelium of testicular feminized (Tfm/y) mice. J Steroid Biochem.

[46] Gao J, Arnold JT, Isaacs JT (2001). Conversion from a paracrine to an autocrine mechanism of androgen-stimulated growth during malignant transformation of prostatic epithelial cells. Cancer Res.

[47] Schaeffer EM, Marchionni L, Huang Z, Simons B, Blackman A, Yu W, Parmigiani G, Berman DM (2008). Androgen-induced programs for prostate epithelial growth and invasion arise in embryogenesis and are reactivated in cancer. Oncogene.

[48] Siiteri PK, Wilson JD (1974). Testosterone formation and metabolism during male sexual differentiation in the human embryo. J Clin Endocrinol Metab.

